# Selective Attention and Concentration Are Related to Lifestyle in Chilean Schoolchildren

**DOI:** 10.3390/children8100856

**Published:** 2021-09-27

**Authors:** Felipe Caamaño-Navarrete, Pedro Ángel Latorre-Román, Juan Párraga-Montilla, Daniel Jerez-Mayorga, Pedro Delgado-Floody

**Affiliations:** 1 Faculty of Education, Universidad Católica de Temuco, Temuco 4780000, Chile; marfel77@gmail.com; 2 Department of Didactics of Music, Plastic and Corporal Expression, University of Jaen, 27301 Jaen, Spain; platorre@ujaen.es (P.Á.L.-R.); jparraga@ujaen.es (J.P.-M.); 3 Faculty of Rehabilitation Sciences, Universidad Andres Bello, Santiago 7591538, Chile; daniel.jerez@unab.cl; 4 Department of Physical Education, Sport and Recreation, Universidad de La Frontera, Temuco 4780000, Chile

**Keywords:** executive function, cognition, dietary patterns, screen time, schoolchildren

## Abstract

The objective of this investigation was to determine the association between selective attention and concentration with physical fitness (i.e., cardiorespiratory fitness (CRF), $\dot{V}$O_2max_, the standing long jump test (SLJ) and handgrip muscle strength (HGS)), lifestyle parameters (i.e., physical activity (PA) level, screen time (ST), sleep duration and food habits) and anthropometric measures (i.e., body mass index (BMI) and waist circumference (WC)) among Chilean schoolchildren. Two hundred and forty-eight schoolchildren (137 boys, 111 girls, 11.80 ± 1.17 and 11.58 ± 1.09 years, respectively) participated. Selective attention, concentration and lifestyle (PA, ST, sleep duration and Mediterranean diet (MD) adherence) were determined using a standard questionnaire. CRF, SLJ, HGS and anthropometric indicators (BMI and WC) were also measured. Selective attention showed a positive association with MD adherence score (β; 5.012, *p* = *p* < 0.05). Concentration was linked inversely to ST (β; −5.498, *p* = *p* < 0.05). Likewise, concentration presented a positive association with MD adherence (β; 2.904, *p* = *p* < 0.05). In conclusion, children’s lifestyles are related to the selective attention and concentration of children; therefore, promoting healthy habits could be a cost-effective strategy in the promotion of cognitive development, as it relates to selective attention and concentration.

## 1. Introduction

School age is a critical stage for cognitive development [1]. In this sense, executive function is a meta-cognitive process necessary for conducting complex and goal-oriented operations [2] that include the follow capacities: inhibitory control, working memory, attention and planning [3]. In this context, executive function plays a fundamental role in children’s learning [4] and is essential for the development of academic skills [5], as evidence has shown that higher levels of executive function are related to better fluid intelligence and success in school [6]. Selective attention allows for the processing of different stimuli [7] by suppressing attention to other distracting stimuli [6]. Recently, empirical evidence has confirmed that it is a crucial element for comprehension and learning processes [8]. It has been indicated that concentration is the capacity to maintain attention with precision [9]. In addition, it has been reported that good concentration improves schoolchildren’s performances on academic tests [10]. In this context, one study showed that attention and concentration are fundamental in cognitive performance [11].

The study of executive function has increased in recent years [12]. A recent study reported that healthy lifestyle factors were positively associated with executive functions in schoolchildren [13]. Likewise, a recent systematic review and meta-analysis indicated that healthy lifestyle factors (e.g., physical activity (PA)) are important for improving executive function [14]. For example, Jirout et al. indicated that although children’s lifestyles have been investigated in the context of health, they are also related with cognition processes in schoolchildren [15]. It has been established that a good lifestyle plays a fundamental role in maintaining cognitive processes into old age [16]. More specifically, the quality of nutrition is a fundamental component of healthy lifestyle behaviours of school-age children [17]. Evidence has shown that a Western diet (i.e., high in refined carbohydrates and saturated fat) may damage brain function [18]. In addition, at this age, the brain’s neuroplasticity is higher; therefore, a low quality of diet may negatively affect the brain’s neurodevelopment and cognitive function [19]. Previous studies have confirmed that dietary habits during childhood are related to learning processes [15] and cognition in schoolchildren [20]. A recent study reported that schoolchildren with better food habits had better cognitive performance [21]. Recently, it was shown that good food habits were positively associated with academic performance [22]. In addition, Allom et al. reported that poor executive function is related to unhealthy eating behaviour [23]. On the contrary, healthy dietary habits, for example, may improve cognitive function and test grades [24]; nonetheless, little is known regarding the relation of diet quality with selective attention and concentration among Chilean schoolchildren [13]. Additionally, more investigations are needed to estimate the effects of healthy food habits on cognitive performance in students [25].

There is a growing interest regarding the impacts of screen time (ST) on cognitive processes [26]. A recent study indicated that a low level of ST was associated with better cognitive function [27]. In this sense, unhealthy lifestyle factors, such as excessive ST, could have negative effects on executive function [28]. Likewise, it has been indicated that ST affects the accuracy of cognitive tasks [29]. Similarly, a previous study has shown that inappropriate ST has negative consequences on cognitive function and other areas related to health [30]; however, a systematic review reported that ST had no negative effects on cognitive development [31]. Therefore, more investigation related to ST and executive function are needed [28]. Along this same line, it is important to evaluate the association between a healthy lifestyle and selective attention and concentration. In addition, to the best of our knowledge, no other investigation has analysed the association of selective attention and concentration with ST recommendations and good food habits in Chilean schoolchildren.

The objective of this investigation was to determine the association between selective attention and concentration with physical fitness (i.e., cardiorespiratory fitness (CRF) classification, $\dot{V}$O_2max_, the standing long jump test (SLJ) and handgrip muscle strength (HGS)), lifestyle parameters (i.e., physical activity (PA) level, ST, sleep duration and food habits) and anthropometric measures (i.e., body mass index (BMI) and waist circumference (WC)) among Chilean schoolchildren. A secondary objective was to compare selective attention and concentration according to ST classifications (<2 h = low ST; ≥2 h = high ST) and Mediterranean diet (MD) adherence levels. 

## 2. Materials and Methods

### 2.1. Participants

**Figure 1:**
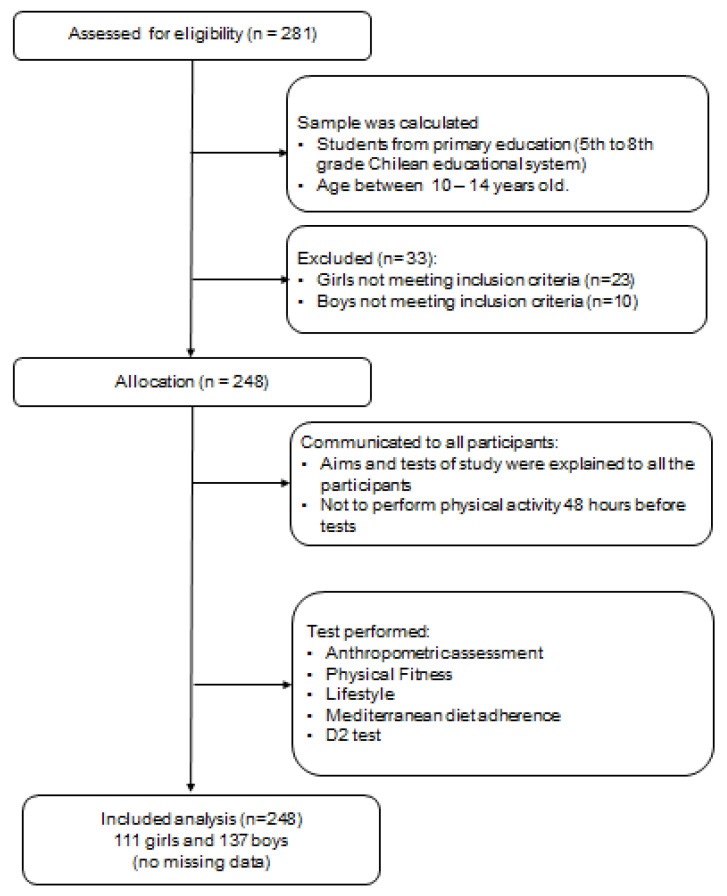
Study design.

This cross-sectional study included 248 schoolchildren (137 boys and 111 girls, 11.80 ± 1.17 and 11.58 ± 1.09 years, respectively) from a subsidised private school in Temuco (Chile). These schools are financed by a mixture of funding from the central government and private contributions [32]. A prior sample size was performed using G*Power software. The following parameters were selected for ANOVA: effect size f = 0.250, α level of 0.05, power level of 0.95, two groups, critical F = 3.886 and a non-sphericity parameter of λ = 13.125. The sample size was determined to be at minimum 210 participants. The sample was determined by convenience. The inclusion criteria were that participants must be Chilean schoolchildren between 10 and 14 years of age and not have any medical conditions or musculoskeletal disorders that might alter their health and physical fitness results. Likewise, schoolchildren with physical, sensorial or intellectual disabilities were excluded. In addition, schoolchildren with missing data and/or who did not present written signed consent were omitted, the study design is shown in Figure 1. The study complied with the Declaration of Helsinki (2013) and was authorised by an Ethics Committee (ABR.19/8.TES Act). The present investigation is part of a doctoral thesis. Informed consent was obtained from all participants. In addition, parents and guardians provided written signed consent for participation.

### 2.2. Measures

#### 2.2.1. Executive Functions

Concentration and attention capacity were obtained using the d2 test [33]. Previous studies have used this test in populations of schoolchildren [9,34]. The d2 consists of a paper and pencil test composed of 14 rows, each with 47 randomly alternated “p” and “d” characters. Each character appears with 1 or 2 dashes placed above and/or below it [8]. Concentration was determined as: number of hits – number of mistakes. Likewise, selective attention was obtained as the number of processed elements – (omissions + mistakes) [34].

#### 2.2.2. Physical Fitness

Cardiorespiratory fitness was determined with the Léger test according to previous indications [35]. The $\dot{V}$O_2max_ (mL/kg/min) was determined using the following equation: $\dot{V}$O_2_peak = 31.025 + 3.238 (V) − 3.248 (A) + 0.1536 (VA), where V is the velocity in km/h achieved at the last stage, and A stands for the participant’s age [35]. High/low CRF was established according to previously determined cut-off points [36].

The SLJT was executed. Each student jumped twice, and the best result was recorded [37]. Handgrip strength was evaluated using a hand dynamometer (TKK 5101^TM^, Grip D; Takei, Tokyo, Japan) according to previously described protocols [38]. The test was performed twice, and the maximum score for each hand was recorded in kilograms. The grip adjustment was made according to the recommendations of Ruiz et al. [39].

#### 2.2.3. Mediterranean Diet Adherence

Food habits were measured by the Krece Plus test, which is based on adherence to the MD. The score from the test was divided as follows: (1) >8, optimal MD; (2) 4–7, moderate; (3) ≤3, very low-quality diet [40].

#### 2.2.4. Levels of Physical Activity

A Physical Activity Questionnaire (PAQ-C) was used to determine the PA levels of the participants. The questionnaire collects information about schoolchildren’s PA over the past seven days [41]. The results for PA were quantified in hours per week.

#### 2.2.5. Screen Time

To evaluate children’s screen time, we used the Krece Plus [42]. This test classifies lifestyle based on the average number of hours spent watching television or playing video games daily. In addition, the participants were divided into two ST groups (<2 h = low ST; ≥2 h = high ST) according to previous indications [43].

#### 2.2.6. Sleep Duration

The Pediatric Sleep Questionnaire was used to determine sleep duration [44]. Parents answered questions referring to the quality and quantity of their children’s sleep.

#### 2.2.7. Anthropometric Assessment

A TANITA scale (model UM–028, Tokyo) was used to measure the children’s weight (kg). The children’s height (m) was calculated with a Seca^®^ stadiometer (model 214, Hamburg, Germany). BMI was calculated following the international formula [45]. A Seca^®^ tape (model 201, Hamburg, Germany) was used to register the waist circumference according to previous recommendations [46].

### 2.3. Procedure

Research assistants visited the selected school during 2019. Parents answered the sleep hour questionnaire during the first two weeks. The evaluations were carried out over four separate sessions by a team of researchers trained in conducting the different tests. CRF, SLJ and HGS were assessed in the first session: prior to the testing sessions, children performed a typical warm-up consisting of 5 min of low-intensity running and 5 min of general exercise. In the second session, anthropometric assessments were carried out in a favourable space facilitated by the school with optimum temperature, reliable privacy and light clothing. Then, lifestyle surveys were applied in the classrooms. A cognitive test was applied in a classroom (final session).

### 2.4. Statistical Analysis

The statistical analyses were developed by SPSS version 21.0 (SPSS Inc., Chicago, IL, USA). The normal distribution was evaluated by the Kolmogorov–Smirnov test. Differences in the comparison between sex, ST and MD adherence groups were determined using an analysis of variance (ANOVA) test. To determine the association between selective attention and concentration and a child’s lifestyle, a simple linear regression and the inclusion of beta (β with 95% confidence intervals (CIs)) were used. The effect size (ES) was calculated using Cohen’s d. Results with a *p* < 0.05 were considered statistically significant.

## 3. Results

Table 1 shows the sociodemographic, anthropometric, lifestyle and executive function characteristics according to sex. Boys reported significantly better scores in $\dot{V}$O_2max_ (mL/kg/min) (*p* = 0.001), HGS right (*p* = 0.001), HGS left (*p* = 0.000), SJL (*p* = 0.000), PA week (*p* = 0.014) and PAC score (*p* = 0.000) than their female peers.

In the association between executive function and lifestyle parameters, in model 0 (not adjusted) and model 1 (adjusted by age and sex), selective attention showed a positive association with MD adherence score (β; 5.612, *p* = *p* < 0.05) and (β; 5.012, *p* = *p* < 0.05), respectively. Concentration had an inverse association with ST h/day in model 0 (β; −5.569, *p* = *p* < 0.05) and model 1 (β; −5.498, *p* = *p* < 0.05). Moreover, concentration showed a positive association with MD adherence score in model 0 (β; 2.864, *p* = *p* < 0.05) and model 1 (β; 2.904, *p* = *p* < 0.05) as seen in Table 2.

**Figure 2:**
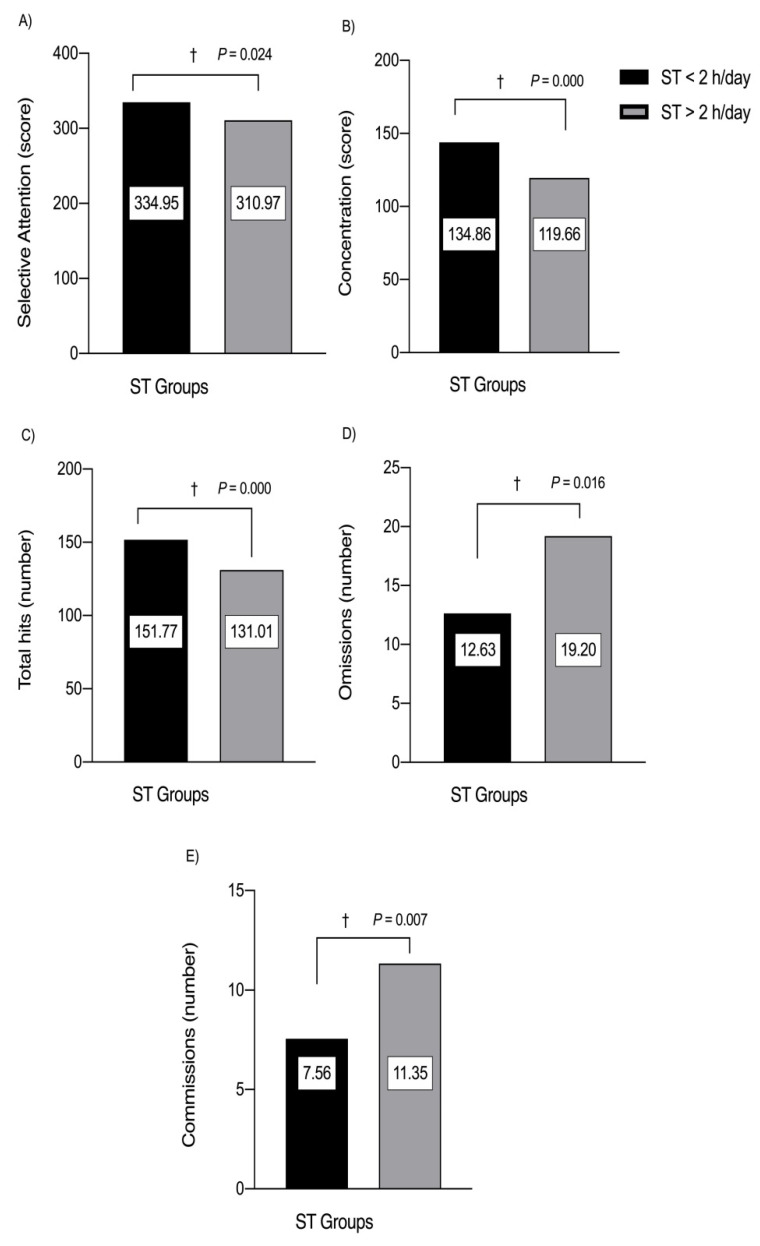
Selective attention (**A**), concentration (**B**), total hits (**C**), omissions (**D**) and commissions (**E**) characteristics in schoolchildren participants by ST groups (<2 h/day/≥2 h/day). (†) Daggers denotes significant differences by group at each respective *p*-value.

When comparing the ST (<2 h/day/≥2 h/day) groups, students with lower STs showed significantly better selective attention (*p* = 0.024), concentration (*p* = 0.000), total hits (*p* = 0.000) and lower omissions (*p* = 0.016) and commissions (*p* = 0.007) in the d2 test than schoolchildren who reported more ST (Figure 2). 

**Figure 3:**
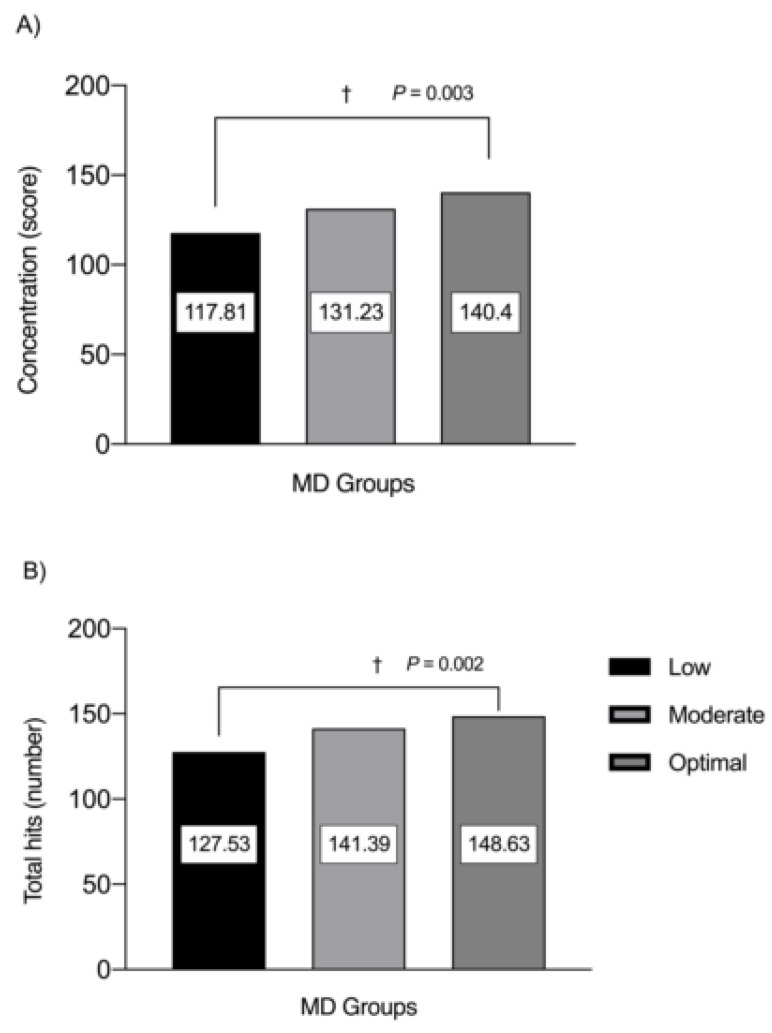
Concentration (**A**) and total hits (**B**) scores in schoolchildren participants by MD groups (low, moderate and optimal). (†) Daggers denotes significant differences by group at each respective *p*-value.

Schoolchildren with optimal MD adherence reported better concentration (*p* = 0.003) and total hits in the d2 test (*p* = 0.002) than moderate and low MD adherence classifications (Figure 3).

## 4. Discussion

The objective of this investigation was to determine the association between selective attention and concentration with physical fitness, lifestyle parameters and anthropometric measures in Chileans students. A secondary aim was to compare selective attention and concentration according to ST classification and MD adherence levels.

The results indicate that (i) selective attention was positively associated with MD adherence (score), while concentration was negatively associated with ST and positively associated with MD adherence (score) in both the unadjusted and adjusted models; (ii) schoolchildren with lower STs showed better selective attention and concentration; (iii) schoolchildren with higher MD adherence scores reported better concentration.

We found that selective attention and concentration were both positively associated with MD adherence (score). Likewise, another investigation conducted among European adolescents reported that higher diet quality scores were linked with attention capacity, and the authors also indicated that dietary patterns were a better determinant of executive function than the analysis of single nutrients [47]. Furthermore, Peña et al. conducted a cross-sectional project with Chilean schoolchildren and reported that students with healthy food habits (i.e., who have a breakfast of high quality) presented better cognitive performance compared with students who did not [21]. Moreover, the findings of another study indicated that poorer food choices were related with reduced performance in verbal and cognitive ability [48]. It has been demonstrated that healthy foods are positively associated with higher performance in executive functions in students [13]. Thus, evidence has shown that a healthy diet, such as having a breakfast, may positively affect cognitive function and school attendance [24]. Additionally, there is strong evidence regarding the impact of nutrition on cognitive function; in this sense, Bellisle [49] indicated that diet can affect cognitive functions in children and adolescents. Similarly, the findings of a systematic review concluded that there was a positive association between good and healthy food habits and executive function [3]. The present results showed that schoolchildren with higher MD adherence scores reported better concentration. Nyaradi et al. [50] conducted a longitudinal study in Australian adolescents and reported that the Western diet score (i.e., characterised by high intakes of take-away food, processed meat and refined food) was related to more total errors in a cognitive test, while students who increased their healthy food intake with fruits and vegetable showed a positive relationship with better cognitive performance. The author of this study also found that having unhealthy food habits at age 14 was associated with poorer psychomotor speed, visual spatial learning and long-term memory performance by 17 years of age. Likewise, Florence et al. [51] showed that specific aspects of diet quality may affect children’s academic performance. Another study reported that a low-quality diet was associated with worse cognitive performance in schoolchildren [52]. In this context, DiGirolamo et al. [53] indicated that it is fundamental to determine nutritional requirements for their possible positive impact on the development of cognitive processes for schoolchildren.

Concentration was negatively associated with ST. In addition, we found that schoolchildren with lower STs showed better selective attention and concentration than their high ST peers. It has been well established that ST is related to different health harms [54]. In addition, there is a growing concern today about the effects of ST on cognition [26]. In this context, Choi and Park [55] reported that there was a correlation between ST and executive function, and that ST mediated the effect on school adjustment through academic performance. A recent study reported that children who never use tablets had significantly better cognitive performance than those who had high STs, with significant differences in prefrontal cortex activation [56]. Likewise, empirical evidence has shown that excessive ST was negatively correlated with the visual word form and the regions related to cognitive control and language; therefore, the authors concluded that limiting ST for schoolchildren was important [57]. Walsh et al. [58] conducted a cross-sectional study with 11,875 American schoolchildren and reported that ST was negatively associated with cognition; likewise, children with high and middle ST classifications had poorer cognition measures than theirs peers in the low classification of ST. Along this same line, another study showed that healthy lifestyle behaviours (i.e., met 60 min of PA, 2 h or less of ST and 9–11 h sleep per night) were associated with better cognition in children [59]. Madigan et al. [60] reported that there was a directional association between levels of ST and child development in a longitudinal study. Likewise, another scoping review indicated that excessive ST was associated with premature cognitive decline and learning problems [61]. Another investigation conducted with Chinese children showed that passive ST (watching TV or videos) was associated with poorer executive function performance and social skills [62]. A recent study conducted in adolescents showed that higher STs were associated with a lower brain derived-neurotrophic factor (BDNF), which can negatively affect cognitive functions and increase the risk factors of neurocognitive deficits [63]. Despite these findings, it has been reported that ST has both negative and positive effects on brain function; therefore, more investigations are needed to clarify the mechanism and possible causal relationships between ST and brain development, especially at ages when brain plasticity is significant [64]. To this end and, contrary to our results, another study reported that smartphone use positively predicted some executive function; therefore, this study indicated that it is important to evaluate the frequency and problematic use of technology rather than ST [26]. Moreover, another study showed that more video game time was positively related to cognition compared with students who played for fewer hours per day [58]. Future studies are needed to clarify the prolonged effects of ST on children’s cognition in different contexts [61].

The limitations of this investigation included its cross-sectional design. In addition, this study selected the sample by convenience. Another limitation was that cognition, food habits and ST results were determined using a written report instrument. In addition, we must consider studying more sociodemographic variables and longitudinal designs to clarify the associations. Likewise, in this study we measured concentration only through the d2 test; therefore, we plan to look for other ways to measure concentration. Moreover, we need to improve the exclusion criteria to limit the sample. Furthermore, as a practical application, it would be important to consider physical activity and educational nutrition interventions in schools to improve executive functions in schoolchildren.

In conclusion, children’s lifestyles were related to the selective attention and concentration of children; thus, the promotion of healthy lifestyle strategies should be prioritised in the education community context. Likewise, healthy food habits, together with decreased ST, could be a cost-effective strategy in promoting cognitive development as it relates to selective attention and concentration.

## Figures and Tables

**Table 1:** Baseline characteristics of the schoolchildren participants by sex at the level of anthropometry, cardiorespiratory fitness, physical activity patterns, concentration and selective attention.

	Total (248)	Girls (111)	Boys (137)	*p*-Value	Cohen’s d
Age (y)	11.70 (11.6, 11.8)	11.58 (11.4, 11.8)	11.80 (11.6, 12.0)	*p* = 0.132	0.193
Anthropometric/body composition					
Body mass (kg)	52.82 (51.3, 54.4)	51.66 (49.6, 53.7)	53.77 (51.5, 56.1)	*p* = 0.183	0.171
BMI	22.06 (21.6, 22.5)	22.14 (21.5, 22.8)	21.99 (21.3, 22.7)	*p* = 0.746	−0.041
WC (cm)	77.57 (76.3, 78.8)	76.70 (75.0, 78.4)	78.25 (76.4, 80.1)	*p* = 0.235	−0.015
Lifestyle/fitness					
Physical activity week (h)	2.39 (2.2, 2.6)	2.16 (1.9, 2.4)	2.58 (2.3, 2.8)	*p* = 0.014	0.154
PAC (score)	28.84 (27.98, 29.69)	26.80 (25.71, 27.89)	30.49 (29.27, 31.70)	*p* = 0.000	
Sleep duration (h/day)	8.48 (8.4, 8.6)	8.48 (8.3, 8.6)	8.48 (8.3, 8.6)	*p* = 0.977	0.003
ST (h/day)	2.90 (2.7, 3.1)	2.99 (2.7, 3.2)	2.82 (2.6, 3.0)	*p* = 0.337	−0.124
MD Adherence (score)	5.96 (5.63, 6.28)	5.85 (5.43, 6.37)	6.05 (5.63, 6.47)	*p* = 0.561	0.078
$\dot{V}$O_2max_ (mL/kg/min)	41.49 (41.0, 42.0)	40.62 (40.0, 41.3)	42.18 (41.5, 42.9)	*p* = 0.001	0.426
HGS Right (kg)	22.13 (21.43, 22.83)	20.77 (19.94, 21.60)	23.21 (22.16, 24.25)	*p* = 0.001	−0.452
HGS Left (kg)	20.33 (19.63, 21.02)	18.73 (17.93, 19.52)	21.61 (20.58, 22.64)	*p* = 0.000	−0.548
SLJ (cm)	120.94 (117.62, 124.26)	108.03 (104.23, 111.83)	131.50 (127.05, 135.95)	*p* = 0.000	1.008
Cognitive Measures					
Selective Attention (score)	321.69 (311.4, 332.0)	314.91 (299.2, 330.6)	327.13 (313.4, 340.8)	*p* = 0.246	0.149
Concentration (score)	130.98 (126.7, 135.3)	127.36 (120.6, 134.1)	133.88 (128.3, 139.4)	*p* = 0.137	0.191
Total attempts (number)	346.99 (336.40, 357.57)	342.80 (327.19, 358.41)	350.35 (335.80, 364.90)	*p* = 0.486	−0.089
Total hits (number)	140.61 (136.47, 144.74)	138.09 (131.85, 144.34)	142.63 (137.06, 148.19)	*p* = 0.284	−0.138
Omissions (number)	15.96 (13.31, 18.62)	17.16 (12.74, 21.59)	15.00 (11.75, 18.25)	*p* = 0.426	0.103
Commissions (number)	9.47 (8.11, 10.82)	10.73 (8.27, 13.19)	8.45 (7.01, 9.90)	*p* = 0.101	0.192

Data are presented as the mean with a 95% confidence interval (CI). *p* < 0.05 was considered statistically significant. BMI = body mass index, WC = waist circumference, ST = screen time, MD = Mediterranean diet, $\dot{V}$O_2max_ = maximal oxygen consumption, HGS = handgrip strength and SLJ = standing long jump.

**Table 2:** Association of selective attention and memory score with sociodemographic, anthropometric, lifestyle and fitness variables in schoolchildren.

		Selective Attention			Concentration		
Outcomes		Beta (95% CI)	*p*-Value	Standardised Beta (SE)	Beta (95% CI)	*p*-Value	Standardised Beta (SE)
Anthropometric variables							
Body weight (kg)	Model 0	−0.451 (−3.18; 2.28)	*p* = 0.745	−0.07 (1.39)	−0.214 (−1.34; 0.92)	*p* = 0.709	−0.08 (0.57)
	Model 1	−0.642 (−3.35; 2.07)	*p* = 0.641	−0.09 (1.379)	−0.169 (−1.28; 0.94)	*p* = 0.765	−0.05 (0.56)
BMI (kg/m^2^)	Model 0	−0.085 (−8.59; 8.43)	*p* = 0.984	0.00 (4.31)	−0.337 (−3.86; 3.18)	*p* = 0.850	−0.04 (1.78)
	Model 1	−0.122 (−8.58; 8.34)	*p* = 0.977	−0.00 (4.29)	−0.511 (−3.98; 2.96))	*p* = 0.772	−0.05 (1.76)
WC (cm)	Model 0	0.536 (−1.90; 2.97)	*p* = 0.664	0.07 (1.23)	0.365 (−0.64; 1.37)	*p* = 0.475	0.11 (0.51)
	Model 1	0.796 (−1.61; 3.20)	*p* = 0.515	0.09 (1.22)	0.408 (−0.58; 1.39)	*p* = 0.417	0.11 (0.50)
Lifestyle							
PA/week (h)	Model 0	1.661 (−8.19; 11.51)	*p* = 0.740	0.03 (4.99)	0.828 (−3.24; 4.90)	*p* = 0.689	0.03 (2.06)
	Model 1	1.797 (−8.07; 11.67)	*p* = 0.720	0.02 (5.00)	0.873 (−3.18; 4.92)	*p* = 0.672	0.03 (2.05)
PAC score	Model 0	−0.508 (−2.49; 1.47)	*p* = 0.613	−0.04 (1.00)	−0.178 (−1.00; 0.64)	*p* = 0.668	−0.03 (0.41)
	Model 1	−0.283 (−2.20; 1.64)	*p* = 0.773	−0.02 (0.97)	−0.113 (−0.90; 0.67)	*p* = 0.778	−0.02 (0.40)
Sleep duration (h/day)	Model 0	−0.794 (−14.40; 12.81)	*p* = 0.909	−0.01 (6.90)	0.908 (−4.72; 6.53)	*p* = 0.751	0.02 (2.85)
	Model 1	−0.313 (−13.87; 13.24)	*p* = 0.964	−0.00 (6.87)	1.133 (−4.43; 6.70)	*p* = 0.689	0.02 (2.82)
Screen time (h/day)	Model 0	−1.179 (−10.59; 8.24)	*p* = 0.805	−0.02 (4.77)	−5.569 (−9.46; −1.68)	*p* < 0.05	−0.21 (1.97)
	Model 1	−0.697 (−10.11; 8.72)	*p* = 0.884	−0.01 (4.77)	−5.498 (−9.36; −1.63)	*p* = 0.006	−0.20(1.96)
MD adherence (score)	Model 0	5.612 (0.63; 10.59)	*p* < 0.05	0.18 (2.53)	2.864 (0.80; 4.92)	*p* < 0.05	0.21 (1.04)
	Model 1	5.012 (0.06; 9.95)	*p* = 0.047	0.15 (2.50)	2.904 (0.87; 4.93)	*p* = 0.005	0.21 (1.03)
Physical fitness							
Léger test (paliers)	Model 0	−216.388 (−454.24; 21.46)	*p* = 0.074	−3.91 (120.59)	7.298 (−91.07; 105.66)	*p* = 0.884	0.31 (49.87)
	Model 1	−2.047 (−23.80; 19.71)	*p* = 0.853	−0.03 (11.03)	4.968 (−3.96; 13.90)	*p* = 0.274	0.21 (4.5)
$\dot{V}$O_2max_ (mL/kg/min)	Model 0	87.690 (−6.20; 181.58)	*p* = 0.067	4.10 (47.60)	−2.935 (−41.76; 35.89)	*p* = 0.882	−0.32 (19.68)
	Model 1	3.287 (−5.00; 11.57)	*p* = 0.435	0.15 (4.20)	−1.683 (−5.08; 1.72)	*p* = 0.331	−0.18 (1.72)
High/low CRF	Model 0	−3.968 (−44.68; 36.74)	*p* = 0.848	−0.02 (20.64)	8.594 (−8.24; 25.43)	*p* = 0.315	0.11 (8.54)
	Model 1	−4.805 (−34.59; 24.98)	*p* = 0.751	−0.02 (15.10)	4.289 (−7.94; 16.52)	*p* = 0.490	0.05 (6.20)
HGS right (kg)	Model 0	0.291 (−4.59; 5.17)	*p* = 0.907	0.02 (2.47)	−0.559 (−2.58; 1.46)	*p* = 0.586	−0.09 (1.02)
	Model 1	0.988 (−3.83; 5.81)	*p* = 0.687	0.06 (2.44)	−0.611 (−2.59; 1.37)	*p* = 0.544	−0.09 (1.00)
HGS left (kg)	Model 0	−1.430 (−6.23; 3.37)	*p* = 0.558	−0.10 (2.44)	0.360 (−1.63; 2.35)	*p* = 0.721	0.06 (1.01)
	Model 1	−1.612 (−6.40; 3.18)	*p* = 0.508	−0.11 (2.43)	0.424 (−1.54; 2.39)	*p* = 0.672	0.06 (0.99)
SLJ (cm)	Model 0	0.269 (−0.36; 0.90)	*p* = 0.400	0.09 (0.32)	0.083 (−0.18; 0.34)	*p* = 0.529	0.06 (0.13)
	Model 1	0.326 (−0.29; 0.95)	*p* = 0.304	0.10 (0.31)	0.093 (−0.16; 0.35)	*p* = 0.473	0.07 (0.13)

The data shown represent beta (95% CI) and standardised beta and standard error (SE). Values of *p* < 0.05 were considered statistically significant. Model 0 = non-adjusted, Model 1 = adjusted by sex and age. BMI = body mass index, WC = waist circumference, PA = physical activity, PAC: physical activity questionnaire, MD = Mediterranean diet, $\dot{V}$O_2max_ = maximal oxygen consumption, CRF = cardiorespiratory fitness, HGS = handgrip strength and SLJ = standing long jump.

## Data Availability

Not applicable.
